# Strengthening local government policies to address health inequities: perspectives from Australian local government stakeholders

**DOI:** 10.1186/s12939-023-01925-3

**Published:** 2023-06-21

**Authors:** Sally Schultz, Christina Zorbas, Anna Peeters, Serene Yoong, Kathryn Backholer

**Affiliations:** Institute for Health Transformation, Deakin University, 221 Burwood Highway, Burwood, VIC 3125 Australia

**Keywords:** Health equity, Health inequalities, Health policy, Local government, Local action

## Abstract

**Background:**

With their close connection to community and increasing preventive health remit, local governments are well positioned to implement policies and programs to address health inequities. Nevertheless, there is a lack of evidence of equity-focused policy action in this sector. We aimed to understand how local government representatives approach equity in the development and implementation of health and wellbeing policies and programs, and to identify potential enablers for strengthening an equity focus.

**Methods:**

We conducted semi-structured interviews (June 2022-January 2023) with 29 health directorate representatives from 21 local governments in Victoria, Australia. Representatives were recruited from urban, regional and rural local government areas, with varying levels of socioeconomic position. Data was analysed inductively using Braun and Clarke’s reflexive thematic analysis, informed by social determinants of health theory and a public policy decision making framework.

**Results:**

Local governments approach health equity in different ways including focusing on priority populations, disadvantaged geographic areas, or by targeting the upstream determinants of health, such as housing and employment. Enabling factors for more equity-oriented local government policy action included those internal to local governments: (i) having a clear conceptualisation of equity, (ii) fostering a strong equity-centric culture, and (iii) developing organisational-wide competency in health equity. External factors related to key stakeholder groups that support and/or influence local governments included: (iv) strong support from community, (v) state government leadership and legislation, and (vi) supportive local partners, networks and NGO’s.

**Conclusions:**

Local governments have a responsibility to implement policies and programs that improve health and reduce health inequities. Local government’s capacity to leverage resources, structures, processes and relationships, internally and across sectors and community, will be key to strengthening equity-oriented local government health policies and programs.

**Supplementary Information:**

The online version contains supplementary material available at 10.1186/s12939-023-01925-3.

## Background

Health is acknowledged as a human right, yet across the world, significant health inequities exist [1]. Such inequities are the result of structural and systemic barriers to health that are unjustly and disproportionately experienced by some individuals and groups [2]. In Australia, while the average life expectancy at birth is one of the highest in the world (83 years in 2020), many groups including Aboriginal and Torres Strait Islander peoples, people living with a disability, people living in rural and remote locations, and those living in areas of socioeconomic disadvantage, experience significantly greater rates of non-communicable diseases and a lower life expectancy [3, 4]. Evidence shows that within countries and across the whole population, the lower a person’s socioeconomic position the worse their overall health, known as the social gradient in health [5]. Over recent decades, there has been a widening of health inequities between the most and least disadvantaged, resulting in a steepening of the social gradient in Australia [6].

Current theoretical models and evidence demonstrate a clear link between health inequities and the unequal distribution of power and resources within political, economic and social systems [5]. Those with more social and economic power enjoy a higher social status and better living and working conditions, which in turn affords them better opportunities to experience good health. In Australia, the socio-political landscape is shaped by systems of colonialism, racism, sexism, and neoliberalism that privilege some, and actively or passively oppress others [7, 8]. These largely invisible systems are perpetuated by policies that promote injustice and exclusion, and diminish power amongst marginalised populations, not only over an individual’s lifetime, but also over generations [8, 9].

Governments have a fundamental responsibility to protect human rights and have considerable influence on political, economic, and social systems that drive health inequities [6, 10]. Consequently, government policy is key to addressing health inequities. Australia has three levels of government: federal, state and local; with each level characterised by a unique, yet interdependent remit for addressing the social determinants of health and health equity [1]. For example, the federal government is responsible for national employment conditions, welfare and universal health care (known as Medicare); state governments are responsible for hospitals, schools, public transport and the justice system; while local governments operate within a particular local jurisdiction or municipality responsible for town planning and development, recreation and cultural services, and local health and community services such as childcare and aged care [11]. Similar to other high-income countries, Australian state and federal governments have increasingly delegated public health functions downwards, albeit often not matched with adequate funding support [13, 19]. This is consistent with the broader literature that suggests that local governments’ strong focus on place-based settings, health and wellbeing and cross-sectoral action, mean they are well positioned to address health inequities [5, 13].

Commitment to taking action on health equity at the local government level is increasingly observed in Australia and other countries, yet, evidence of effective policy action is limited [14–22]. Multiple studies of high-income European countries, using diverse methods including scoping reviews, meta-narrative mapping analysis and ethnographic fieldwork, found municipal-level public policies often problematise equity in narrow and reductionist ways, with a focus on intermediary determinants (such as those targeting health behaviours), rather than targeting the structural (‘causes of the cause’) determinants of health inequities [14–17]. Likewise in Australia, interviews with local government policy-makers across three state jurisdictions found that while some stakeholders acknowledged that health is socially determined, others perceived that health was largely determined by “lifestyle choices” [18, 19]. Furthermore, analyses of municipal health and wellbeing plans and other strategic documents in Victoria found that while ‘health equity’ and the ‘social determinants’ were included, representations were often rhetorical in nature (for example, describing the social determinants, reporting data related to priority populations, and setting goals to reduce inequities) and did not necessarily inform policy action [20–22]. To advance action on health equity will therefore require an understanding of the barriers and enablers of considering health equity in local government policy action.

There is limited international research exploring enablers and/or barriers to effective local public health policy action on health inequities. In the UK and Europe, strong political commitment to addressing health inequities; policy processes and budget provisions to support implementation; whole-of organisation focus on equity; and supportive national and regional level governments were found to facilitate equity-oriented local government policy action [23, 24]. The international literature also cites lack of local-level equity-related epidemiological data, and lack of evidence to inform policy action as barriers to progress. Despite the significant opportunity local governments have to help reduce the gap between those who experience the most and least disadvantage, and level up (reduce the steepness) of the social gradient for all Australians, no studies have explored how to strengthen equity in local government policy action in Australia. To address this gap and build on the international literature, this study aims to understand perceptions of local government representatives on the approaches local governments take in *developing, prioritising and implementing policies and programs* (herein referred to as ‘*policy action’*) to reduce health inequities, and identify potential enablers for strengthening their equity focus.

## Methods

### Study design

We conducted a qualitative descriptive study, using in-depth semi-structured interviews with local government representatives (i.e., employees of local governments) in Victoria, Australia to explore two research questions: (i) how does local government consider and approach health equity in policies and programs? and (ii) what factors may enable local governments to strengthen their approach to health equity?

Qualitative methods allowed for an exploration of perspectives from local government representatives, while also acknowledging the background of the researchers [25]. The research team has extensive expertise in policy research to improve population health and health equity in the Australian context, and recognise the role of government and broader society in either contributing to, or helping to redress, the unequal distribution of power and resources that underlie the determinants of health inequities.

### Theoretical framework

Our data collection and analysis were informed by the World Health Organization (WHO) *Commission on Social Determinants of Health conceptual framework (2010) and* Howlett and Ramesh’s (2003) public policy decision making framework [26, 27]. The CSDH was developed to help orient government and societal action towards comprehensively addressing the social determinants of health inequities, including structural factors (such as policies, systems and social institutions), and intermediary factors (such as material, psychosocial, behavioural and biological factors) [26]. The CSDH was used to help understand how local government representatives conceptualised and attempted to address the determinants of health inequities. Howlett and Ramesh’s (2003) framework identifies five stages for producing public policies: agenda setting, policy formulation, adoption (or decision making), implementation and evaluation [27]. This framework was used to highlight different stages of the policy making process where equity could be strengthened.

### Sample and recruitment

The 79 local government areas (LGAs) in Victoria were categorised according to area-level remoteness (Greater Melbourne or regional/rural) and by area-level socioeconomic position (SEP) (higher/lower) [28, 29]. The latter were identified using the Index of Relative Socio-economic Disadvantage (IRSD) quintiles (using 2016 census data) where quintiles 1, 2 and 3 represented lower SEP areas and quintiles 4 and 5 represented higher SEP areas [29]. Recruitment was prioritised to ensure diverse representation across the strata, and continued until local governments representatives from all LGAs were invited.

Every four years, Victorian local governments are required to develop a plan that identifies “goals and strategies for creating a local community in which people can achieve maximum health and wellbeing” [30]. Accordingly, Victorian local government representatives involved in the development and/or implementation of these plans were deemed suitable to answer the research questions.

An email introducing the study was sent to all 79 Victorian local governments, marked to the attention of a senior manager within the directorate responsible for community health and wellbeing. Contact names were obtained from publicly available information on local government websites, or through snowball sampling. If recipients were interested in the study they could choose to participate themselves or nominate another representative from within their local government. Written or verbal consent was provided by all participants and included a statement that they had authority to discuss their organisational policies and processes.

### Data collection

Semi-structured interviews were conducted by the first author between June 2022 and January 2023 by videoconference (Zoom or Microsoft Teams). Participants were firstly asked to describe their role and tenure, then asked a series of question to understand how they defined equity and health equity, how their local government approached equity in the development and implementation of health policies and programs, and the factors that could strengthen equity in policy action (see Additional file 1 for interview guide). Average duration of the interviews was 55 min. Interviews were audio and video recorded with participants’ permission and transcribed verbatim. Participants were given the opportunity to review a copy of the transcript.

### Data interpretation

Data (i.e. interview transcripts) were managed in NVivo 12 (IQR International software) and analysed inductively using Braun and Clarke’s six steps of reflexive thematical analysis [25]. In step one, interview transcripts were read and re-read, noting initial thoughts about how participants understood equity, how they perceived equity was approached within their local government and key barriers and enablers to equity-oriented policy action. Step two involved reading the transcript again and generating codes, guided by the research questions and theoretical frameworks. The first two interviews were dual-coded by the first and second authors, who then met to discuss codes and emerging ideas. The first author then completed the coding and constructed themes from the codes (step 3), before meeting with the broader research team to review and workshop key themes (step 4). These themes were refined further to ensure they were clear, succinct and answered the research questions (step 5). Finally in step 6, the findings and themes were described in the drafting of the manuscript, supported by illustrative quotes [25]. Throughout the data collection and interpretation process, the first author engaged in reflexive practice by taking and discussing notes after each interview with the research team. These discussions centred around developing a deep familiarisation with the results, and carefully considering findings in relation to the research team’s own understanding of key health equity, public health and policy making concepts. This process of reflection and ongoing dialogue contributed to the development and refinement of our themes.

## Results

Twenty-nine representatives from 21 local governments participated in semi-structured interviews. Participants worked in manager/team leader and officer-level roles within the directorate responsible for community health and wellbeing. Role functions included department leadership (5), health and social policy and planning (11), strategic health projects (4), health promotion and community development (9). Participating local governments were from diverse geographic areas. Approximately half were from metropolitan areas (11 metropolitan, 7 regional, 3 rural) and just over 60% were from areas of low SEP (13 from IRSD quintiles 1–3, 8 from IRSD quintiles 4–5).

The following themes describe how local government approach health equity in policy action and how their approach can be strengthened.

### 1. Local governments’ approach to equity in policy action

Participants agreed that local governments had a critical role to play in addressing health inequities. Local government’s role as a major employer, its direct connection and regular consultation with community, and local government’s power and remit to support community health and wellbeing underpinned this perspective. Participants believed these characteristics gave local governments a unique opportunity to build health equity knowledge within their organisation, advance health equity advocacy efforts in the broader community, and role model equitable health policy action to partners and local organisations.

Councils (also known as local governments) are in a position where they can dictate a lot of what’s happening out in the community. We’re developing programs and we’re sending out resources to community members. So, yes, we should be in a position where we are talking about equity across the board.

– Local government representative from low SEP, metropolitan area.

Participants’ understanding of equity was generally well aligned to current theories and evidence [13, 26, 31]. For example, participants understood health inequities to mean that not everyone in the community has the same opportunity to live life to their fullest potential. Key concepts discussed by participants included the social determinants of health, identifying priority groups who are more likely to experience structural barriers to health and the importance of policies and programs to support people proportionate to their needs. Intersectionality was also highlighted by a few participants as an emerging concept that is guiding the way they approach health equity.

For me equity is that strong intersectional focus applied at various stages across a project or a priority area. So it’s that planning to have that intersectional focus of inclusion in terms of who’s at the table? Who are you having those discussions with? But also in terms of the project at the ground level. Who are you recruiting? Who are you involving in the actual decision making? But also in the doing and the delivery of the projects as well.

– Local government representative from low SEP, metropolitan area.

Overall, perceptions of the extent and nature of equity-oriented local government policy action varied. Several participants expressed that while health equity-related goals and frameworks were included in local government strategic plans, they did not necessarily translate into policy action. Where participants described health equity actions that were being taken, the most common actions included targeting programs in geographic areas of socioeconomic disadvantage, addressing a specific need (e.g. food relief), or actions to support priority group/s (e.g. Aboriginal and Torres Strait Islander people, those living with disability, and/or those living in public housing). Conversely, for a few local government representatives, policy action was designed to address the broader social/structural determinants of health (e.g. employment, education and racism) for the whole population, rather than targeting specific health priorities or priority groups. Regardless of the perceived level of action, all participants suggested that their local government needed to do more to address health inequities in their community.

### 2. Enablers to strengthen local governments’ approach to health equity

**Fig. 1:**
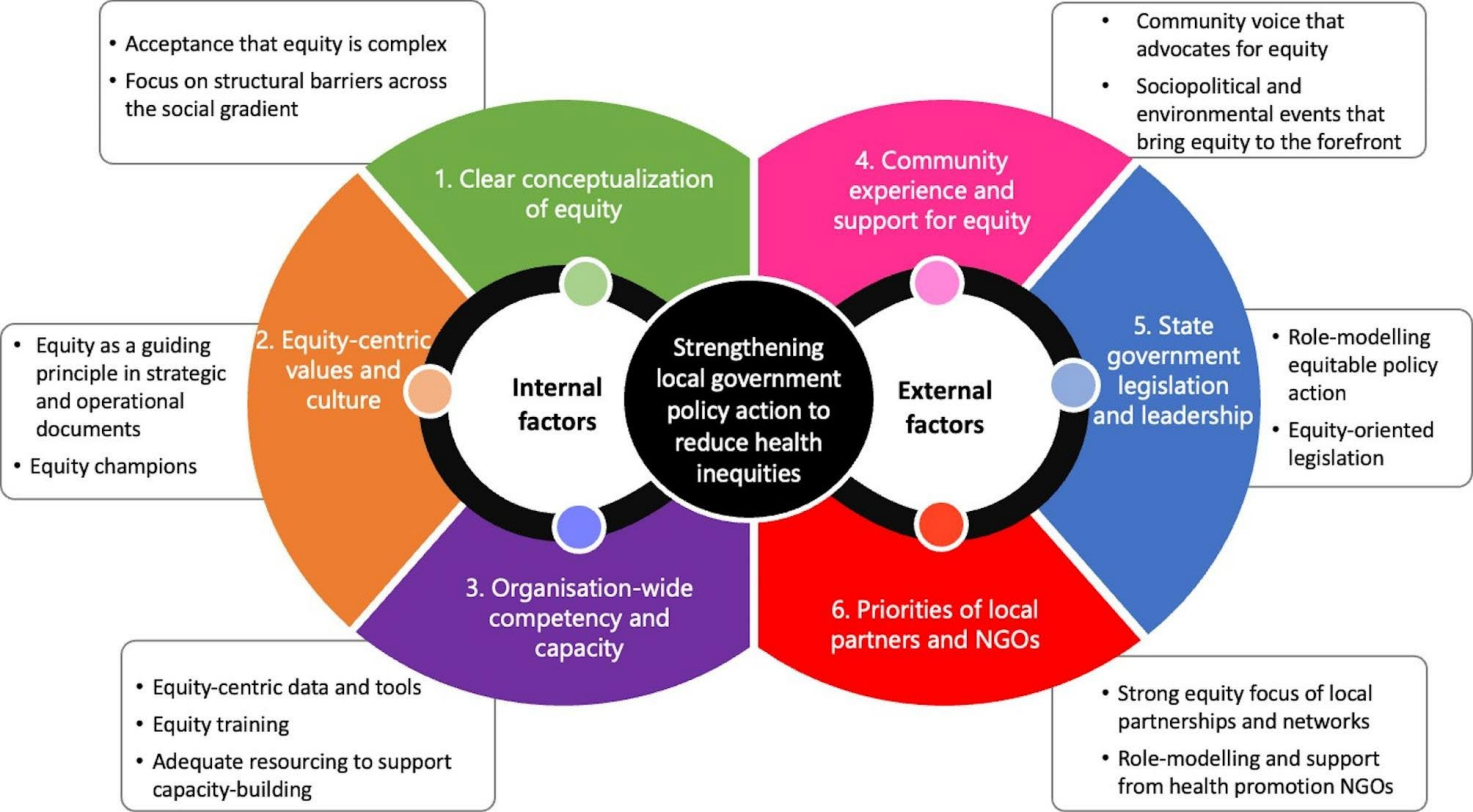
Enablers of equity-oriented local government policy action: perspectives from Australian local government stakeholders

Six key themes were identified that described enabling factors of equity-oriented local government policy action. Three themes were considered internal factors (those within the control of local governments) and three themes related to key external stakeholders. As illustrated in Fig. 1, major themes (within main diagram) and sub-themes (rectangular boxes) are depicted in a figure eight model, highlighting the interconnectedness between themes.

## Internal factors

### Enabling factor 1: clear conceptualisation of equity

#### Acceptance that equity is complex

Equity was often described by participants as a complex issue which was not well understood across local governments. A general unfamiliarity with language associated with equity including the word ‘equity’, as well as ‘social determinants’, ‘priority populations’ and ‘intersectionality’ was flagged as potentially stalling action on health equity.

*“Like planners who speak a completely different language so if you try to go to them and talk your language they just look at you and say ‘what, it sounds too hard I’m not doing it’”.*

– Local government representative from high SEP, metropolitan area.

Some participants believed the perceived complexity around equity could be partly addressed through staff training, or by using more familiar terms such as ‘fairness’ or ‘equal opportunity’. However, it was acknowledged that equity is a person-centred concept, and because people are multifaceted and complex, it is important to accept that addressing health inequities is going be multifaceted and complex. Taking a long-term, intersectional approach that avoided over-simplifying equity was deemed critical for equitable policy action.

#### Focus on structural barriers across the social gradient

A historical policy focus on more downstream determinants of health, such as behaviour change programs, may have influenced how equity is conceived within some local governments. A shift in focus towards systemic and structural root causes of health inequities was considered key to both understanding and acting on health inequities. This may include a range of direct actions on the social determinants of health (e.g. actions that support or complement state and federal government actions to improve determinants such as employment, education, housing), as well as indirect action through thoughtful design and implementation of health and other policy actions (e.g. targeted programs for priority groups or via universal programs that aim to act across the whole social gradient).

*“I think the latest cost of living crisis is going to really highlight that* (health inequities*), because people who never thought that they would struggle are now going to struggle. And…you don’t want to wait for people to slide so far down the social gradient that then they hit that welfare sweet spot. We need to be thinking about them along the whole gradient”.*

– Local government representative from high SEP, metropolitan area.

### Enabling factor 2: equity-centric organisational culture

#### Equity as a guiding principle in strategic and operational documents

Centering equity as a core value of the organisation was described as an important enabler of equity-oriented local government policy action. Representatives who reported working at local governments that incorporated ‘equity’ in their values or guiding principles, or had formally endorsed a social equity framework, perceived that this helped to embed equity in health policy decisions, even in the absence of equity-focused decision making tools. For example, one representative said that when their team considered new proposals and funding requests, one of their key criteria is “how does it align with our guiding principles?” If equity was well-considered, the policy or program was more likely to be prioritised.

Furthermore, it was perceived that purposeful equity-related language in job titles, such as ‘health equity and evaluation officer’ or ‘health equity and communications officer’, as well as in position descriptions can contribute to an equity centred culture.

*“It even gets down to the shaping of your PDs (position descriptions), and trying to bring this to the surface when you’re recruiting people. And it’s almost a value, so if it’s not in your value statement, I think it’s always very hard to get traction. Because your PDs move into your performance and your performance review, and your education and training, and all of that. I mean, I think that’s a really strong flow on”.*

- Local government representative from low SEP, regional area.

#### Equity champions

Equity champions were also seen as important drivers of a health equity focus across the organisation. Equity champions were people from any part of the organisation who formally or informally advocated for the importance of health equity and priority populations. These people often held roles as councillors, CEO and executive leaders, specialist roles focused on priority populations (e.g. Disability Inclusion Officer, Aboriginal Liaison Officer, Social Inclusion Officer) or other staff within the community health and wellbeing directorate.

Coming into the organisation, I was very much aware of a very strong social justice premise. There have been leaders that have come and gone that carried that through, which helps craft and create a culture. And there’s also been some really strong councillors who are democratically elected leaders that have carried that badge as well.

– Local government representative from low SEP, regional area.

Participants also commonly noted that equity champions often had health promotion, public health, social work or similar qualifications and backgrounds, giving them knowledge, skills and/or experience in understanding and addressing inequities. The more senior the role, the more influence the equity champion was perceived to have in fostering an equity-centric culture and helping to embed equity considerations in policy processes.

It was widely acknowledged that local governments generally work in functional silos, and the extent to which different departments believed they were responsible for addressing health inequities varied (often viewed to be the remit of the community health and wellbeing directorate only). Participants suggested equity champions, embedded across local government organisations can help in breaking down functional silos in relation to health equity and build a sense of responsibility for equity in policy action across all functions.

### Enabling factor 3: organisational-wide competency and capacity

#### Equity-centric data and tools

Equity-relevant data was a fundamental facilitator for the development and prioritisation of equitable policy action. Population census data disaggregated by key equity indicators was readily accessible and used primarily to identify priority populations and the geographic areas and settings for targeted interventions. Participants discussed commonly using data related to area-level socioeconomic disadvantage, English proficiency, Aboriginal or Torres Strait Islander peoples, single-parent households, and people living with a disability, as well as place-based data including indexes for community socio-educational advantage and children’s development vulnerability. Where resources were available, this was often supplemented by local-level data collection such as community health surveys. Conversely, many participants discussed a lack of equity-oriented evaluation data to inform policy action as a significant gap. Policy and program evaluations were reported as being rarely prioritised, inadequately resourced, and outcome data were seldom disaggregated by equity indicators.

*“I guess shaping the way that you word your targets is important. Say the program or event isn’t going to be about getting the most bums on seats, it’s going to be about engaging from people from X, Y and Z groups. And trying to not just tick off that we’ve got one Aboriginal person, one multicultural person, one socioeconomically disadvantaged person, but make the targets about ‘have we engaged well with those groups rather than getting a large number of people’.*

– Local government representative from low SEP, regional area.

Whilst it was perceived that equity-focused evaluations of policies and programs could help inform future actions, some participants described their local government as having “…*pretty strict rules around what (data) we are, and are not, allowed to gather and use*” and were unsure as to the extent to which they would be able to collect sociodemographic data that would allow for meaningful equity-focused evaluations.

While the participants’ use of equity-related tools was low, the majority believed tools to help staff consider equity when planning and implementing policies would be beneficial. Tools such as equity-focused health impact assessments, that could be applied to all local government policies and programs (beyond health and wellbeing remit) and were supported by training and equity champions were viewed as having the most potential impact. Almost all participants indicated their local government had recently developed or were developing a Gender Equality Impact Assessment (GIA), as required by the Victorian Gender Equality Act 2020 [32]. Given this is a legislated gender-oriented equity-oriented tool, it opened up discussions about whether the GIA could be expanded to incorporate a broader concept of equity. Some thought a single tool would be preferable as it would be more user-friendly, while others raised concerns about potentially diluting the quality of the GIA.

#### Equity training

The majority of participants suggested that equity training was essential to building knowledge, developing skills and fostering an equity-centric mindset. Even participants who perceived their local government had a strong equity focus believed “*there’s still a very strong educative piece that’s required*” both within the community health and wellbeing directorate and across the broader organisation. Training was described as being most beneficial when it was delivered regularly across the whole organisation, was designed to helped people understand health and social equity, the social determinants of health, intersectionality, and the specific barriers experienced by priority groups, and was delivered by people with lived experience of inequities.

*“Training has to have people with lived experience of the equity barrier you’re talking about. If you just have somebody come in and talk about everyone should be equal and we should have this, that, and the other and it’s from a theoretical point of view, people don’t pay that much attention to it.“*

- Local government representative from low SEP, regional/rural area.

One local government representative suggested that coordinating equity training topics (e.g. formal workshops or lunchtime information sessions) with publicised community focus weeks (e.g. Elder Abuse Week, National Reconciliation Week) could help build capacity in equity on an ongoing basis.

#### Adequate resourcing to support capacity building

Participants expressed the importance of having adequate resources to support an equity focus over time. Some participants considered their local government to be fairly well-resourced within the community health and wellbeing directorate to focus on equity, particularly those with dedicated staff responsible for health and social planning and projects, health promotion, community development, and social equity leadership roles. For other local governments, particularly those from low socioeconomic, regional / rural areas, participants reported scarce human resources available within the community health and wellbeing directorate, and often no resources attributed to specialist priority population roles or equity-related roles. These local governments experienced challenges developing and implementing health and wellbeing policies and programs in general, so resourcing policies and programs with an equity lens was considered out of reach. Furthermore, participants discussed the importance of being given the time and resources to *do equity well*. When roles have a mandate to help address social and health inequities, it was deemed critical that those roles be given time and resources to connect, collaborate and consult meaningfully across local government and with external stakeholders including the community, local partners and other community organisations, and networks.

I’d love to spend more time with other departments to learn exactly what it is that they do. Because I know the principles in my head, but unless I know exactly what they do, I can’t really give them any specific advice around how to implement it.

– Local government representative from low SEP, regional area.

## External factors

### Enabling factor 4: community experience and support for equity

#### Community voice that advocates for equity

The community was described as a key informant for local government policy action via formal and informal consultations and community advocacy. Accordingly, when community voice prioritises equity, local governments were viewed as more likely to also prioritise equity-focused policy.

*“One of the focus areas that came up* (in community consultations) *was around, caring for all community members, and so we hear really strongly from the community as well that that is important”.*

– Local government representative from high SEP, metropolitan area.

Several participants expressed concern that consultations are a “biased activity” whereby the “*loudest voices*” are often people who have social and economic power and resources, and do not represent those who disproportionately experience health inequities, or who are “*grappling with Maslow’s Hierarchy of Needs in their day-to-day*”. To combat this, several participants spoke of the importance of strong governance processes designed to contain the influence of the ‘loudest voices’, coupled with deliberate efforts to connect and listen to those that bring lived experience of social and/or economic exclusion.

#### Sociopolitical and environmental events that bring equity to the forefront

Sociopolitical and environmental events (e.g. strong COVID-19 restrictions imposed in Victoria, the current cost of living crisis, and major flood or fire events experienced in many regional areas) often triggered intermediary local government action to addresses health inequities, for example, by supporting local emergency responses to deliver financial, food and other social relief services. It was perceived that such events helped to raise public awareness of the need to take action on the social determinants that make people vulnerable to the impacts of such events (e.g. insecure employment, food insecurity, and inadequate transport to food retail and healthcare).

It’s interesting how COVID has really triggered things, where people and society can sort of handle a certain amount of inequity, and it’s kind of accepted. But when it tips a bit further, people start to take notice. Maybe it’s the number of people and the people that they didn’t think would be in that position.

– Local government representative from high SEP, metropolitan area.

Building on the awareness created by such events, proactive engagement by local governments with community about health and social equity, and role-modelling equity-oriented action was discussed as having great potential to generate or amplify community support and advocacy for equity in local communities.

### Enabling factor 5: state government leadership and legislation

#### Role-modelling equity in policy action

State government was considered highly influential in setting the agenda on health equity-related issues that local governments can “*piggy-back on*” or “*take on some of that shared responsibility*”.

I think the Victorian State Government’s doing a brilliant job, I really do. I’ve never seen it so progressive ever really. I think that they’ve decided, “Look, we’re just going to really try and achieve some of the really big complex issues,“. And that takes time, and that takes grit, and that takes leadership, and nerve.

– Local government representative from low SEP, regional area.

Examples cited by participants included the Yoorrook Justice Commission (formal truth-telling commission into past and ongoing injustices experienced by First Nations People as a result of colonisation) [33], leadership in gender equality [32], and investment in social housing [34]. Participants also suggested there was an opportunity for State government to include meaningful equity-centric criteria in applications for grants and funded initiatives.

#### Equity-oriented legislation

State government legislation was considered to be one of the most important enablers of local government action: “*it’s at the top of the tree in terms of getting stuff done*”. In particular, all participants spoke about the impact that the Gender Equality Act 2020 had on local government priorities and resources [32]. For example, local governments are now required by the Act to conduct GIAs on all policies, programs and services that can have a significant public impact [32]. As such local governments have invested resources to develop and implement training, tools and human resource support (to varying extents), which many participants believed would not have been realised without the State legislation.

We couldn’t have gotten as far as we have with the gender impact assessments without it being a requirement that Council has to comply with.

- Local government representative from low SEP, metropolitan area

Some participants were unsure of the State government’s appetite for legislation to address other forms of inequity, and what this could look like in practice. However, one avenue being explored by some local governments was to broaden the scope of the existing gender impact assessment tool to consider inequities caused by other social indicators including socioeconomic position, race and ethnicity and geographic location.

### Enabling factor 6: equity focus of local partners and health promotion networks

#### Strong equity focus of local partnerships and networks

Local organisations, including district health services, Aboriginal Community Controlled Health Organisations (ACCHOs), local multicultural organisations and other local government partners, were seen as critical drivers of equity-oriented local government policy action through two mechanisms. First, these organisations help to inform policy development and prioritisation by representing the voices of communities that experience health inequities during local government consultations and network meetings. Second, local governments often lean on local partners for implementing local government policies, so have considerable influence on how equity is considered during policy or program implementation.

We have a health and wellbeing partnership that meets quarterly and I think there’s 30 people from probably 20 different organisations. Basically anyone that’s working in the health and community fields. Representatives from the hospital, health services, Neighbourhood Houses, various multicultural groups and the local women’s health agency. It’s a big forum for discussing the issues that everyone’s seeing and keeping everyone updated with opportunities for partnership and networking.

- Local government representative from low SEP, regional/rural area

#### Role-modelling and support from health promotion non-government organisations

Non-government organisations (NGOs) such as the Victorian Health Promotion Foundation (VicHealth), the Heart Foundation and Cancer Council were identified as a trusted source of information regarding health promotion. Some participants perceived that these organisations had potential to show greater leadership by developing and sharing equity-focused health promotion resources and by incorporating meaningful equity criteria in grant applications (similar to State government).

“*Because we know they’ve done all the research, and the background, and they come from a trustworthy source… so if we go back to our councillors and they don’t like it, we say, “well actually VicHealth have said it*””.

- Local government representative from low SEP, regional/rural area

## Discussion

We found that local government representatives in Victoria, Australia predominantly share the view that local government policy has a critical role to play in addressing health inequities. Whilst there was considerable variation in the perceived extent and nature of what constitutes equity in policy action, interviewees consistently reported that local governments can strengthen their current approach to achieve equitable health outcomes. Enabling factors for more equity-oriented local government policy action included factors internal to local governments: (i) having a clear conceptualisation of equity, (ii) fostering a strong equity-centric culture, (iii) developing organisational-wide competency in equity. External factors were related to key external stakeholder groups that support or influence local government action: (iv) the community, (v) State government and (vi) local partners, networks and NGO’s.

Our findings that local government health and wellbeing representatives perceive that they have a key role to play in addressing health inequities corresponds with international literature that suggests that local-level action has significant potential to influence the social determinants of health [35–38]. As a major employer, community leader, designer of place-based initiatives and provider of health and well-being services, local governments have great opportunities to drive and support local-level action on health inequities. This also aligns with community views, with a recent study finding 91% of the Australian public surveyed believed local governments should deliver services that contribute to a healthier and fairer society [39].

Previous studies exploring health equity in local- and national-level policies have found that while equity is often acknowledged in strategic documents, policy actions typically lacked an equity-orientation and rarely focused on upstream social determinants [20–22, 40–44]. Our study is consistent with this, whereby we found that while some local governments oriented policy action towards addressing upstream determinants either across the whole population or targeted actions for specific priority groups, many actions either addressed more downstream determinants (e.g. supporting food relief efforts) or did not attempt to address health inequities at all. This may be partly due to the remits of the three levels of government, where federal and state government can have a greater influence on social determinants such as income, housing and education [11]. Action on the broader social determinants of health is also generally less politically palatable or well understood than actions that target behaviour and lifestyle [45]. This is despite the well accepted notion that reducing health inequities will require a commitment to addressing upstream determinants of health [26, 36].

Several of the perceived enablers to equity-oriented policy action found in our study have also been reported in the international literature. For example, having clear equity-oriented strategic goals, adequate resourcing, access to equity-focused epidemiological and evaluation data and taking a whole-of-organisation approach [23, 24]. Our results also echo previous studies on the influence of national and regional levels of governments and local partner organisations in facilitating improved action on equity at the local government level. Our study extends this prior work by demonstrating the inter-related nature of these enabling factors, which is highlighted in the following policy implications.

### Implications for local governments

We found that equity is viewed as a complex issue, which risks action on health inequities being put in the ‘too hard basket’. Standard public sector responses to complexity have been viewed as inadequate, with leaders opting for ‘quick fixes’, instead of the multifaceted, inclusive responses required to address the ‘complex causes of complex problems’ [46]. A paradigm shift that encourages local governments to *lean into* the complexity of equity, avoiding both policy over-simplification and inaction is an important first step towards equity in local health policy action. Principles of ‘complexity leadership’ offer one approach to enabling such a paradigm shift [47]. Complexity leadership challenges those with positional power to think about systems and multiple causal loops to problems, to let go of the notion of always ‘knowing what to do’, and focus on being adaptive rather than linear in developing and implementing solutions [47]. Resources and frameworks can also be used to help make sense of the complexity to the broader organisation. United Nations (UN) Women is an example of an organisation that recognised that equity is complex and developed a resource guide to help work through the complexity using an intersectionality framework [32]. Other frameworks such as the WHO’s *Commission on Social Determinants of Health conceptual framework* [26], VicHealth’s *Fair Foundations framework for health equity* [48] and more recently, the *Nutrition Equity Framework* [49], can also help unpack the complexity by helping local governments identify entry points for policy action across different levels and types of social determinants. Contemporary evidence and theory also suggests that to level-up the social gradient (rather than simply narrow the health gap), local governments should consider policy action that is universal (accessible to all) and implemented with a scale and intensity that is proportionate to the level of disadvantage (known as proportionate universalism) [36]. For example, local government action to enhance usage and accessibility of local parks or community gardens for all, combined with specific outreach and engagement with those experiencing health and social inequities, to increase their use of the spaces.

Situating health equity as a *process* and an *outcome* can help to centre action towards shifting the ideologies, beliefs, structures, systems and processes, that contribute to the determinants of health inequities. Our study identified a number of enablers that support a process-centred approach for local governments, such as building equity-focused strategic principles, champions, tools and training, and listening to the community voice. Whilst previous studies have found that equity in strategic documents was largely rhetorical with little influence on actual policy action [40, 41], we found that if supported by equity champions in leadership positions, equity-focused strategic goals and principles can act as a high-level mechanism for staff to apply an equity lens, even in the absence of an explicit equity-focused policy decision making tool.

There is evidence to suggest that pragmatic and inconsistent approaches to equity in health policy-making hinders the translation of equity-related policy objectives to effective policy action [50]. We found that equity-focused tools fostered consistency and accountability in the way equity was considered in health policy action, while also providing an opportunity for ‘on the job’ professional development, especially when implementation of a tool is supported by equity champions or experts. The use of health equity tools is widely recommended by the WHO and other leading health promoting agencies [26, 51–53]. While the international literature includes a substantial number of health equity tools, research has shown that for tools to be effective, they need to be practical, user-friendly, adaptable to diverse contexts, and help to build practitioner competency within the setting [54]. As such, equity tools are often tailored for specific contexts or organisations [55–59]. Our study highlighted different stages of the policy development process where equity tools and frameworks could be used to ensure a strong equity lens is applied when prioritising and implementing local government health policies. These include during the (i) prioritisation of health policy actions, (ii) development of specific health policies, programs and services (e.g. using an equity impact assessment), and (iii) reviews of implementation plans (e.g. annual review of local government action plans). In Victoria, the Gender Equality Act (2020) requires all local governments to conduct a Gender Impact Assessments (GIA) of all policies, programs and services that are new or up for review and have a direct and significant impact on the public [60]. Given this tool is already mandated, State and/or local government could explore expanding the tool to include broader conceptions of equity. This may first require an evaluation of the acceptability, feasibility and impact of current equity-focused tools for local governments. Finally, inclusion of equity-related criteria in local government grant applications may also support a stronger equity focus within proposed health programs that are implemented by local partner organisations. The Health Research Council of New Zealand is one example of an organisation with strong equity criteria, requiring all grant applicants to demonstrate how their proposal is likely to advance Maori health [61] .

Our findings further highlighted the role that ongoing equity training can play in helping teams outside the health directorate understand equity and their potential roles in addressing health inequities. There is evidence that training on the social determinants of health helps to develop knowledge about the root causes of health inequities and emphasises the importance of addressing them at the community level [62, 63]. Equity-related skills and attitudes are also essential for public health and policy professionals to be advocates for change [64]. We also found equity-competency and capacity needed to be developed across the whole organisation, which is also consistent with evidence that action on the social determinants cannot be limited to those working in health, with all departments having a role to play to address inequities [65].

We found that measuring and monitoring health equity outcomes is also essential for achieving health equity. National census-based data was readily accessible to local governments, and used to help plan and prioritise strategies, policies and programs. However, few local governments had the resources to measure and monitor health inequities or the social determinants that cause them at a local-level, representing an important research and policy gap. Likewise, the lack of equity-focused policy and program evaluations found in our study has also been reported as a key barrier to enabling equity-oriented policy action in the literature [23]. A range of tools and frameworks are available to inform equity in data collection and evaluation activities [66, 67]. In particular, measures that recognise equity as both a process and an outcome will be important, including measuring changes to staff knowledge and skills, changes to internal processes and structures, as well as changes in health and social outcomes.

Conceptualisations of health equity as a *process* to build widespread community capacity to identify and tackle causes of health inequities, highlights the need for strong, equitable community engagement and participation in policy decisions. Our study found that community voice was a key component of equity-oriented policy action, but required strong governance processes to contain the influence of the “loudest” and often more resourced voices. A core tenet of effective health and social policy design to address equity at the local level is authentic and ongoing participation of community with lived experience of the barriers to health equity [68]. Yet, the lived experiences of many core equity groups remain unheard, with policy-makers often determining what is best for communities, while minimising their self-determination and agency [69]. This may be due to inadequate understanding of the definitions and methods that support good community participation, a lack of cultural safety and allyship, and existing long-standing institutional tensions with policies and processes that are underpinned by colonial neoliberal ideologies [70]. For governments to disrupt this status quo and commit to equity-oriented policies and processes, power and resources will need to be redistributed towards those who are most affected by health inequities. Greater investment in participatory platforms that enable diverse communities to prioritise and address their health needs is one way local governments can redistribute power and foster meaningful community participation in decision-making [71].

### Implications for upper tiers of government and NGOs

Whilst local governments are well-positioned to support actions that can positively influence health inequities, including actions that target the social determinants of health, they must be adequately supported by equity-oriented policy action and funding support from upper tiers of government. In the UK, local government’s ability to address the increased expectations and responsibilities for health equity have not been matched with funding support [38]. Likewise, our study and others found adequate resourcing underpins local government’s capacity for equity-oriented policy action [23, 24]. In response to the post-pandemic cost-of-living crisis, a new rate cap (where the Victorian State Government set a limit on the revenue local governments can collect from their community to fund local services) has been applied to all Victorian local governments, putting more pressure on limited resources, especially in disadvantaged rural/regional areas [72]. Increased implementation support from upper-tiers of government, and NGO’s is needed to meet the increased expectations of local governments to address health inequities. Such support is also aligned with a proportionate universal approach, where universal action is implemented at a local- level proportionate to the need [13].

## Implications for local community organisations

Our study highlighted the critical importance of local organisations, including district health services and other community organisations, that partner with local governments to develop and implement equity-oriented health policies and programs. It is widely acknowledged that intersectoral action between governments and non-government entities focused on upstream social determinants is a key approach to improving health equity [6, 9, 73]. The body of evidence evaluating intersectoral action on health equity is scarce, with a systematic review finding that where evaluations did exist, they were methodologically weak and showed moderate to no effect on the social determinants of health [74]. In Australia, research suggests intersectoral action often favours individual behaviour approaches, and may reflect ‘lifestyle drift’, where policy action starts off with a commitment to address the wider social determinants of health, but drifts downwards to lifestyle interventions designed to shift individual behaviours [75, 76]. Identifying and learning from cases where local intersectoral action has had positive equity outcomes could benefit both local governments and local community organisations.

## Strengths and limitations

To our knowledge, this study is the first to describe specific enablers of local government policy action to reduce health inequities. A key strength of the study is that diverse perspectives across metro and rural/regional areas and lower and higher SEP areas in Victoria were represented in our sample. As participants were recruited from the directorate responsible for community health and wellbeing, they were generally knowledgeable and interested in health equity. Future research could explore perspectives from stakeholders, including councillors, executive leaders and other departments, particularly given the potential influence of the broader local government organisation on equity-oriented policy action. This study was underpinned by a strong theoretical basis, grounded in social determinants of health equity theory, and supported by a policy decision making framework to identify where and how equity can be considered throughout policy decision processes. Taking a reflexive approach that acknowledged the positionality of the research team, strengthened the richness and transparency of data analysed. Nevertheless, future research would benefit from engaging other perspectives in interpreting the findings, including those from local government and community. In Australia, local government priorities and actions are strongly linked to state and territory government priorities, so findings related to Victorian State government may not be applicable to other jurisdictions in Australia or internationally. Finally, it should also be noted that whilst both barriers and enablers to equity-oriented policy action were explored in interviews, we chose to frame the results in the positive (where barriers were reframed as enablers) to inspire positive action.

## Conclusion

Local governments have a key role to play in shifting systems and structures at the local level to address social and health inequities. Local government’s capacity to leverage resources, structures, processes and relationships, internally and across sectors and community, will be key to strengthening equity in local government policies and programs. Building local government competency and capacity for equity-oriented policy action will require supportive policies and resourcing from upper-tiers of government and health-promoting NGOs.

## Electronic supplementary material

Below is the link to the electronic supplementary material.

Supplementary Material 1

## Data Availability

The datasets used and/or analysed during the current study are available from the corresponding author on reasonable request.

## Abbreviations

GIA: Gender Impact Assessment
LGA: Local Government Area
NGO: Non-Government Organisation
SEP: Socioeconomic position
WHO: World Health Organization
