# Supplementary material for: Strengthening local government policies to address health inequities: perspectives from Australian local government stakeholders
Source: Int J Equity Health. 2023 Jun 21;22:119. doi: 10.1186/s12939-023-01925-3 (PMC10283264; doi:10.1186/s12939-023-01925-3)
Supplement: Supplementary file 1 — Supplementary Material 1 [file 12939_2023_1925_MOESM1_ESM.docx]

**Additional File 1**

**Interview Guide: Equity in local government policies and programs**

**Introduction**

Q1. To start, could you please tell me about your role?

*(Prompts: tenure, department / team, type of involvement with local government health and healthy eating policies and programs?)*

Q2. Could you tell me about the key policies or programs your council has to promote nutrition and healthy eating?

*(Prompts: (food procurement policy, education program)*

Q3. Can you describe how your council prioritises which policies and programs are ­­selected?

*(Prompts: Who is involved, key drivers for choosing which policies to implement / criteria,*

*processes, frameworks or tools that are used?)*

**Equity in healthy eating policies**

Q4. When you think about health equity, what does it mean to you?

Q5. What approach does your local government take to address health equity?

*(Prompts: Priority groups, level of understanding / focus your council has on addressing health inequities, local governments role in addressing inequities)*

Q6. What sort of impact do you think current your policies have on your priority equity groups? Why?

*(Prompts: Do you evaluate your healthy eating policies and programs for their impact on priority equity groups? - If yes, how did you measure that?)*

Q7. Can you describe any processes or tools that your council uses to help them consider equity

*(Prompts: Data, Health Impact Assessments, lived experiences)*

**Barriers and Enablers**

Q8a. What factors within council do or could support an equity-focus when your healthy eating policies and program are developed and implemented?

Q8b. What factors make it challenging to consider equity?

*(Prompts: org structure, culture, compatibility, priority, incentives, leadership, resources, knowledge, readiness, equity champion, knowledge, beliefs, skills)*

Q9a. What external factors help you consider equity?

Q9b. What external factors make it challenging to consider equity?

*(Prompts: community organisations, state/federal govt policies, other LG actions)*

Q10. What strategies or tools would practically help you ensure your policies and programs address health inequities?*.*

*(Prompts: - tools, information, training, working with other local government portfolios, community organisations, other groups)*

Q11. We’ve covered a lot today, but is there anything else you would like to add?
